# Overexpression of GitrL in Recombinant Rabies Virus rLBNSE-GitrL Enhances Innate Immunity by Activating Dendritic Cells and Innate Immune-Related Pathways and Genes

**DOI:** 10.3390/v17101354

**Published:** 2025-10-09

**Authors:** Yufang Wang, Xiao Xing, Zhimin Xiong, Yong Wang, Yaping Liu, Yingying Li

**Affiliations:** 1 College of Basic Medicine, Dali University, Dali 671000, China; 15587038350@163.com (Y.W.); a17861606528@icloud.com (X.X.); 19116217657@163.com (Z.X.); ayxwy76@163.com (Y.W.); 17717107839@163.com (Y.L.); 2 Dali Nursing Vocational College, Dali 671000, China

**Keywords:** recombinant rabies vaccine, GitrL, innate immunity, dendritic cells, innate immune-related signaling pathways, innate immune-related genes

## Abstract

Rabies, a zoonotic infectious disease causing central nervous system inflammation, remains a threat to public health in regions with limited medical resources. Vaccination effectively reduces rabies incidence and mortality, underscoring the need for vaccines that are cost-effective, immunogenic, protective, and safe. This study constructed a recombinant rabies virus (rRABV)-overexpressing glucocorticoid-induced tumor necrosis factor receptor ligand (GitrL), named rLBNSE-GitrL, using a reverse genetic operating system. rLBNSE-GitrL exhibited similar in vitro phenotypic characteristics and immune safety as the parent RABV (rLBNSE). This recombinant virus stimulated the production of a greater number of activated dendritic cells (DCs) compared to rLBNSE. The enhanced innate immune response induced by rLBNSE-GitrL may be mediated through the activation of innate immune-related signaling pathways, such as the tumor necrosis factor (TNF), and chemokine signaling pathways, and the upregulation of a series of innate immune-related genes, including MMP2, IL-6, CXCL9, TIMP1, IL-17d, and TNF-α. Consequently, rLBNSE-GitrL elicited significantly higher levels of RABV vaccine-induced virus-neutralizing antibodies (VNA), IgG, and IgM compared to rLBNSE as early as 3 days post-immunization (dpi), thereby improving the protective effect in mice. Collectively, the overexpression of GitrL facilitated the induction of early and potent antibody responses following RABV immunization.

## 1. Introduction

Rabies is an acute zoonotic disease caused by rabies virus (RABV), which leads to the programmed death of nerve cells after inflammation [1,2]. RABV is a non-segmented, negative-sense, single-stranded RNA virus, classified within the Lyssavirus genus of the Rhabdoviridae family [3]. Annually, RABV is responsible for approximately 59,000 human fatalities, predominantly in regions of Asia and Africa with inadequate healthcare infrastructure [4]. Transmission to humans occurs in over 99% of cases via dog bites or licks. Due to the absence of effective treatment, rabies exhibits a near 100% mortality rate in humans [5]. Effective prevention measures can significantly reduce the morbidity and mortality associated with this disease [6]. Vaccination remains the primary strategy for rabies prevention and control. While the currently available inactivated rabies vaccine is safe, it is costly, and its immunogenic efficacy is suboptimal [7]. In contrast, live-attenuated recombinant rabies virus (rRABV) vaccines have the potential to elicit protective immunity after a single-dose administration, positioning them as promising candidates for an ideal rabies vaccine.

Glucocorticoid-induced tumor factor receptor ligand (GitrL), the principal ligand for Glucocorticoid-induced tumor factor receptor (Gitr), is a 20 kDa type II transmembrane protein within the tumor necrosis factor family. It shares 20% sequence homology in its extracellular domains with other tumor necrosis factor (TNF) ligands [8]. GitrL is expressed in antigen-presenting cells (APCs) like dendritic cells (DCs), macrophages, and B cells [9]. Interactions between Gitr and GitrL activate DCs and subsequently stimulate T lymphocyte activation [10]. When exogenous pathogens invade the body, they are recognized by pattern recognition receptors (PRRs), such as Toll-like receptors (TLRs), RIG-I-like receptors (RLRs), and NOD-like receptors (NLRs). These receptors, located on the surface or within the cytoplasm of innate immune cells like DCs, recognize pathogen-associated molecular patterns (PAMPs), triggering downstream signaling in the innate immune pathway [11]. These downstream signaling factors include those associated with NLRs, mitogen-activated protein kinases (MAPKs), TLRs, and their downstream components such as TNF, extracellular signal-regulated kinase (ERK), c-jun N-terminal kinase (JNK), p38 kinase (p38) [12], and nuclear factor-kappa B (NF-kB) [13], and these factors are integral to inflammation and innate immune responses [14]. Nonetheless, the capacity of rLBNSE-GitrL to augment innate immunity via activated DCs and the GitrL-associated signaling pathway remains largely unexplored.

This study constructed a rRABV expressing the mouse GitrL protein, designated rLBNSE-GitrL, and evaluated its impact on the innate immune response in a mouse model. The results demonstrate that overexpression of GitrL enhanced early antibody production against RABV infection by activating DCs and upregulating innate immune-related signaling pathways and genes.

## 2. Materials and Methods

### 2.1. Cells, Viruses, Antibodies, and Animals

The BSR cells were cloned from BHK-21 cells and were cultured in Dulbecco’s modified Eagle’s medium (DMEM) (Gibco, Grand Island, NY, USA) with 10% fetal bovine serum (FBS) (Gibco, Grand Island, NY, USA) and antibiotics (100 µg/mL Penicillin and 100 µg/mL Streptomycin) (Beyotime, Wuhan, China). The parent strain rLBNSE was developed from the SAD-b19 virus by removing its pseudogene, incorporating BsiWI and NheI sites between the G and L genes, and introducing mutations at positions 194 and 333 of the G protein [15]. The rabies challenge virus CVS-24 was propagated in the brains of suckling ICR mice. Antibodies obtained from Biolegend (San Diego, CA, USA) were used for flow cytometry analysis of DCs, including FITC-CD11c (clone N418), APC-CD80 (clone 16-10A1), PE-CD86 (clone GL-1), and PE-MHCII (clone M1/42). To detect rRABV-specific antibody haplotypes, HRP-coupled goat anti-mouse IgG and IgM antibodies from Boster (Wuhan, China) were utilized. All animals were sourced from SPF Biotechnology Co., Ltd. (Beijing, China). The rRABV-protective efficacy experiments adhered to the Huazhong University of Science and Technology Science Ethics Committee’s protocol (Protocol No. [2024]IACUC:4696). Other animal experiments followed the protocol of the Dali University Science Ethics Committee (Protocol No. 2022-SL-89). All procedures were performed in accordance with the ARRIVE guidelines.

### 2.2. Construction and Rescue of rRABV Expressing GitrL in Mouse

The GitrL gene was amplified from the brain of a rabies-infected mouse using RT-PCR. The primers for GitrL amplification were forward primer 5′-TTG CGT ACG ATG GAG GAA ATG CCT TTG-3′ and reverse primer 5′-CTA GCT AGC CTA AGA GAT GAA TGG tag-3′, with BsiWI and NheI sites underlined. The GitrL gene was inserted between the G and L genes of the vaccine vector pLBNSE, producing the generation of pLBNSE-GitrL. Full-length infectious clones (pLBNSE-GitrL) and four helper plasmids expressing the N, P, G, and L genes of rLBNSE were transfected into BSR cells using SuperFect transfection reagent (Qiagen, Valencia, CA, USA) according to procedures described in previous studies [15] The successful rescue of rLBNSE-GitrL was verified using FITC-conjugated RABV N protein-specific antibodies.

### 2.3. Virus Titration

The virus titer in BSR cells was quantified using a direct fluorescent antibody assay. Serial 10-fold dilutions of the rLBNSE-GitrL were added to 96-well plates containing BSR cells. The plates were incubated at 37 °C with 5% CO_2_ for 48 h. Post incubation, the cells underwent three washes with 200 µL of phosphate-buffered saline (PBS), fixation with pre-cooled acetone for 15 min, followed by another three PBS washes and air-drying. The cells were then stained with FITC-conjugated antibodies specific to the RABV N protein and incubated for 45 min. Fluorescent foci indicating antigen presence were enumerated as FFU/mL using an Olympus X51 fluorescence microscope (Olympus, Tokyo, Japan).

### 2.4. Genetic Stability Assay

The rLBNSE-GitrL was successfully rescued and defined as the first passage. BSR cells were infected with rLBNSE-GitrL at a multiplicity of infection (MOI) of 0.01, incubated at 37 °C for 1 h, and then maintained in fresh medium at 34 °C for 4 days. The virus titer in the cell culture supernatant was determined. DNA was extracted from the infected cells, amplified using specific primers (forward: 5′-GTG GGG GTG AGA CCA GAC-3′, reverse: 5′-TAG ACC TCT CCA GGA TCG-3′), and sequenced. This process was repeated for 10 consecutive passages.

### 2.5. Determination of rLBNSE-GitrL Growth Curve

The BSR cells were seeded at a density of 1 × 10^5^ cells per well in 24-well plates and incubated overnight at 37 °C with 5% CO_2_. Then, the cells were infected with rLBNSE or rLBNSE-GitrL at MOI of 0.01 and 5. After 1 h of incubation, the cells were washed with PBS, and fresh DMEM supplemented with 2% FBS was added. The cultures were then transferred to a 34 °C incubator. Culture supernatants were collected at 1, 2, 3, 4, and 5 days post infection to measure viral titers. Viral growth curves, including both multi-step and single-step, were plotted based on the observed titers at these time points.

### 2.6. Cell Viability Assay

Cells were inoculated in a 96-well plate at a density of 1 × 10^4^ cells per well and exposed to rLBNSE or rLBNSE-GitrL at a MOI of 0.01 for 1 h. Post-infection, the cells were cultured in DMEM supplemented with 2% FBS and antibiotics. Cell viability was assessed at 1, 2, 3, and 4 days post-infection using cell proliferation assay kits (Promega, Madison, WI, USA) following the manufacturer’s protocol.

### 2.7. GitrL Concentration Determination by ELISA

BSR cells were seeded in 24-well plates at a density of 2 × 10^5^ cells per well and incubated overnight at 37 °C with 5% CO_2_. Cells were then infected with rLBNSE or rLBNSE-GitrL at MOI of 1, 0.1, 0.01, and 0.001. The expression of GitrL in the supernatants of infected cells was quantified using a Mouse GitrL ELISA kit (RayBiotech, Peachtree Corners, GA, USA), following the manufacturer’s instructions. Absorbance was measured at 450 nm using a Spectra MAX 190 microplate reader.

### 2.8. Average Fluorescence Spot Density

The BSR cells were infected with rLBNSE or rLBNSE-GitrL at an MOI of 0.01. The infected cells were then incubated at 34 °C for 24 h and 48 h in DMEM containing 1% low-melting-point agarose to immobilize the cell culture supernatant. The agarose was subsequently removed, and the infected cells were stained with FITC-conjugated RABV N protein-specific antibodies. The size of the resulting fluorescent aggregates was then compared using fluorescence microscopy.

### 2.9. Pathogenicity of rLBNSE-GitrL

Six-week-old female ICR mice (n = 7) were inoculated intracerebrally (i.c.) with 30 μL of a solution containing 5 × 10^6^ FFU of rLBNSE, rLBNSE-GitrL, or mock infected with DMEM under isoflurane anesthesia. Five-day-old ICR mice were i.c. injected with 100 FFU of different rRABVs in 10 μL DMEM. The weight and survival rate of adult female mice were monitored for 2 weeks. The survival rates of 5-day-old ICR suckling mice were checked daily for 3 weeks. Total RNA was extracted from the brains of adult ICR mice infected with rRABVs for 3 days, and the expression levels of RABV N gene mRNA, viral genome RNA, and GitrL mRNA were detected by RT-qPCR.

### 2.10. Reverse Transcription Quantitative PCR (RT-qPCR)

Total RNA was extracted from brain or muscle tissues and subjected to reverse transcription quantitative PCR (RT-qPCR). 1 µg of RNA was reverse transcribed into complementary DNA (cDNA) using a first-strand cDNA synthesis kit (Toyobo, Osaka, Japan). Quantitative PCR was performed using a one-step SYBR green RT-qPCR mix kit (Toyobo, Osaka, Japan). Standard curves were constructed for each viral target using threshold cycle (Ct) data plotted against the log copy numbers of serially diluted plasmid standards carrying the RABV N gene. The log copy numbers of viral messenger RNA (mRNA) and viral genome RNA (vRNA) were normalized to 1 µg of total RNA. The mRNA levels were normalized to β-actin mRNA levels. The primer sequence is shown in Table 1.

### 2.11. Flow Cytometry Assay

Flow cytometry was employed to detect DCs both in vitro and in vivo. In the in vitro experiments, bone marrow cells were isolated from BALB/c mice, filtered through a 40 μm cell mesh, and cultured in RPMI 1640 medium supplemented with 10% FBS, 20 ng/mL recombinant mouse granulocyte–macrophage colony-stimulating factor (GM-CSF), and 10 ng/mL interleukin-4 (IL-4) at a density of 2 × 10^5^ cells/mL for 6 days. These cells were then seeded in 6-well plates (10^6^ cells/mL) and infected with either rLBNSE or rLBNSE-GitrL at a MOI of 0.01 for 24 h prior to flow cytometry analysis. In the in vivo experiments, three BALB/c mice in each group were immunized with 100 μL of 10^6^ FFU of rLBNSE, rLBNSE-GitrL, or DMEM as a mock. In vivo, groups of three BALB/c mice were immunized with 100 µL of 10^6^ FFU of rLBNSE, rLBNSE-GitrL, or DMEM as a mock. Inguinal lymph nodes (LNs) cells were collected on days 3 and 6 post-immunization and prepared as single-cell suspensions. The cells were washed with PBS, centrifuged, and then incubated with 0.2% bovine serum albumin (BSA) and fluorescently labeled antibodies for 30 min at 4 °C in the dark. After two washes with PBS containing 0.2% BSA, the resuspended cells (1 × 10^5^ cells per sample) were analyzed by flow cytometry using a BD FACSCalibur instrument (BD, Piscataway, NJ, USA).

### 2.12. IFN-α and IL-12p40 in DCs Supernatant Were Detected by ELISA

Following stimulation with rRABVs, the supernatant from bone marrow-derived dendritic cells (BMDCs) was centrifuged at 4 °C for 10 min. The expression levels of IFN-α and IL-12p40 in the supernatant were detected using mouse-specific ELISA kits for IFN-α and IL-12p40 (NeoBioscience Technology, Beijing, China) in accordance with the manufacturer’s instructions. Concentrations were measured with a Spectra MAX 190 microplate reader.

### 2.13. Virus-Neutralizing Antibody (VNA) Test

Blood samples were collected from mice at designated timepoints after immunization, and virus-neutralizing antibody (VNA) levels were determined using the Fluorescent Antibody Virus Neutralization (FAVN) assay. Briefly, 50 μL of serially diluted experimental samples and standard sera (National Institute for Biological Standards and Control, Herts, UK) were added to a 96-well plate. Subsequently, 50 μL of a CVS-11 virus suspension containing 100 FFU was added to each well, and the plates were incubated at 37 °C and 5% CO_2_ for 1 h. Finally, 50 μL of BSR cells (4 × 10^5^ cells/mL) were added, and the plates were incubated at 37 °C. Following a 2-day incubation at 5% CO_2_, samples were stained with FITC-conjugated anti-RABV N antibodies for 45 min at 37 °C and subsequently washed 3 times with PBS. Fluorescence was examined using an Olympus X51 fluorescence microscope (Olympus, Tokyo, Japan). The fluorescence intensity of the prepared serum sample was compared to that of a standard sample to determine the VNA titer, expressed in international units per milliliter (IU/mL).

### 2.14. rRABV-Specific Antibody Subclass Test

The RABV-specific antibody subclass was detected and analyzed using ELISA. 96-well ELISA plates were coated with 100 µL of Na_2_CO_3_ buffer (5 mm, pH 9.6) containing RABV G protein (0.5 µg/well) (Boster, Wuhan, China) and incubated at 4 °C for 12 h. Following this, the plates were washed 3 times with PBS-Tween and blocked with 5% skim milk buffer at 37 °C for 2 h. Serum samples from immunized mice were diluted in PBS-Tween at a 1:30 ratio, added to each well, and incubated at 37 °C for 2 h. After incubation, samples were washed 3 times with PBS-Tween and incubated with HRP-conjugated goat anti-mouse immunoglobulins (IgG) (1:1000) and IgM (1:2000) (Boster, Wuhan, China) for 45 min at 37 °C. Following another three washes with PBS-Tween, they were incubated with 100 µL tetramethyl benzidine (TMB) substrate (Boster, Wuhan, China) in the dark at 37 °C for 30 min. The reaction was halted by adding 2M H_2_SO_4_ (Boster, Wuhan, China). Absorbance at 450 nm was measured using a Spectra MAX 190 microplate reader.

### 2.15. Protective Efficacy Test

The six-week-old female ICR mice (n = 10) were inoculated intramuscularly (i.m.) with 100 µL of a solution containing 10^6^ FFU of rLBNSE or rLBNSE-GitrL, or mock inoculated with DMEM. On 3 dpi, 50 × lethal dose 50 (LD50) of CVS-24 was injected i.c. into the three groups of mice. Survival of the mice was continuously monitored and recorded for 20 days, and the data were analyzed using statistical methods.

### 2.16. Transcriptome Sequencing (RNA-Seq)

The study utilized 6–8-week-old BALB/c mice, which were randomly assigned to three groups. Each group received a 100 μL intramuscular injection of either 10^6^ FFU of rLBNSE, rLBNSE-GitrL, or DMEM (as mock). Muscle tissue from the immunization site was harvested 6 days post-immunization and subjected to transcriptome sequencing. Total RNA was extracted from the muscle samples, and 1 μg per sample was used to construct the initial sequencing library by Hieff NG Illumina Biotechnology (Shanghai) Co., Ltd. (Yeasen Biotechnology, Shanghai, China). Quality control and sequence alignment of the raw Fastq data were then performed. The present study leveraged a comprehensive suite of bioinformatics tools and databases, including NCBI, Pfam, KOG/COG, Swiss-Prot, KO, GO gene homology database, and Nr, to conduct detailed gene sequence comparison and functional annotation. Transcript abundance was quantified using the FPKM metric, and key differentially expressed genes were validated via RT-qPCR analysis, the sequence design of the primer is shown in Table 1. Differential expression was assessed across biological replicates, and pathway enrichment analysis was performed using the GO database.

### 2.17. Statistical Analysis

All data were analyzed by GraphPad Prism 9.5.0 software (GraphPad Software, Shanghai, China). Kaplan–Meier survival curves were analyzed by the log-rank test for the percent survival experiments. Significant differences between the groups in two independent experiments were determined by an unpaired two-tailed *t*-test (parametric test) and were indicated at three levels: *, *p* < 0.05; **, *p* < 0.01; and ***, *p* < 0.001.

## 3. Results

### 3.1. Characterization of rRABV Expressing GitrL

**Figure 1:**
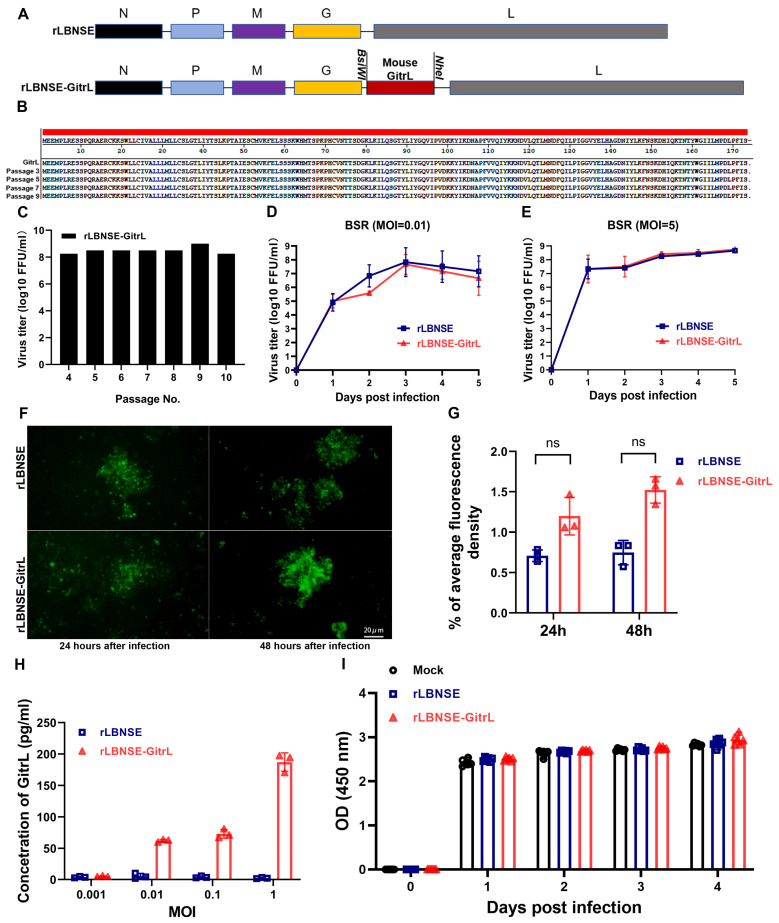
Characteristics of rRABV expressing GitrL in vitro. (**A**) Schematic diagrams for the construction of rLBNSE and rLBNSE-GitrL. The vector pLBNSE was derived from the SAD-B19 strain with pseudogene deletion. *BsiW*I and *Nhe*I were introduced between the *G* and *L* genes. *N*, *P*, *M*, *G*, and *L* represent RABV nucleoprotein, phosphoprotein, matrix, glycoprotein, and polymerase genes, respectively. (**B**) Amino acid sequence alignment of the *GitrL* gene, the red area indicates the amino acid sequence was consistent with the reference sequence of the *GitrL* gene. (**C**) Viral titers from the fourth passage to the tenth passage on BSR cells. (**D**,**E**) The virus titers of different rRABVs-infected cells with MOI = 0.01 and MOI = 5 were determined at 1, 2, 3, 4, and 5 dpi, respectively. On this basis, the multi-step growth curves (**D**) and one-step growth curves (**E**) of rLBNSE- and rLBNSE-GitrL-infected BSR cells were plotted. (**F**,**G**) Morphology and size of viral fluorescent spots formed by different rRABVs in BSR cells. (**H**) The expression level of GitrL in the supernatant of infected cells was detected by ELISA. (**I**) Cell viability of different rRABVs-infected cells with MOI = 0.01 at 1, 2, 3, and 4 dpi, respectively. The error bar represented the mean ± standard deviation (SD, n = 3). The following symbols are used to indicate significant differences between groups: ns, not significant.

The role of GitrL in RABV-induced immune responses was investigated by cloning the mouse GitrL gene into rLBNSE vectors, generating the rLBNSE-GitrL (Figure 1A). The rRABV demonstrated consistent GitrL sequence (Figure 1B) and stable titration (Figure 1C) across ten consecutive passages in BSR cells. The replication kinetics of rRABVs harboring the exogenous GitrL gene were evaluated in BSR cells. Viral growth was assessed through multi-step (Figure 1D) and single-step (Figure 1E) growth curve analyses following infection at a low MOI of 0.01, and high MOI of 5, respectively. The results demonstrated no significant differences in viral titers between the rRABVs, indicating that the insertion of the exogenous GitrL gene did not substantially alter virus replication and proliferation in vitro. The viral fluorescent spots formed by rLBNSE-GitrL and rLBNSE exhibited similar morphology and size in BSR cells (Figure 1F,G). This suggests that the insertion of the GitrL gene did not significantly affect the replication and spread of the rabies virus in infected cells. ELISA results demonstrated that BSR cells infected with rLBNSE-GitrL expressed GitrL in a dose-dependent manner, while rLBNSE-infected cells did not (Figure 1H). Furthermore, cell viability assays revealed no significant differences in the growth and viability of BSR cells infected with rLBNSE-GitrL compared to those infected with rLBNSE, indicating that the expression of GitrL did not have adverse effects on the infected cells (Figure 1I).

### 3.2. Pathogenicity of rRABV-Overexpressing GitrL

**Figure 2:**
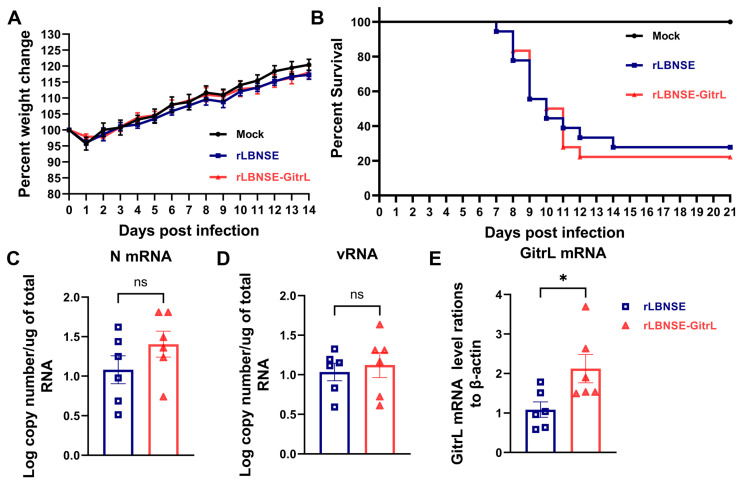
The pathogenicity of rRABV expressing GitrL. (**A**) Body weight changes in female 6-week-old ICR mice (n = 7) infected with 5 × 10^6^ FFU of rLBNSE, rLBNSE-GitrL, or DMEM via i.c. pathways. (**B**) Survival rate of 5-day-old mice (n = 18) i.c. injected with 100 FFU of the above rRABVs. (**C**) N mRNA levels, (**D**) vRNA levels, (**E**) GitrL mRNA levels in the infected BALB/c mouse brain (n = 6) were quantified using RT-qPCR. The error bar indicates the mean ± standard error of the mean (SEM). The following symbols are used to indicate significant differences between groups: *, *p* < 0.05; ns, not significant.

The safety of rRABV is of paramount importance. To evaluate the potential adverse effects of rLBNSE-GitrL, we conducted an animal study. Three groups of 6-week-old female ICR mice were inoculated with rLBNSE (5 × 10^6^ FFU), rLBNSE-GitrL (5 × 10^6^ FFU), or DMEM (as mock), respectively. Body weight and survival rate were monitored continuously for 14 dpi. None of the mice exhibited any clinical symptoms associated with rabies. The weight curves of rLBNSE-GitrL-infected mice were not significantly different from those of rLBNSE-infected mice (Figure 2A). Three groups of 5-day-old suckling ICR mice were injected with 100 FFU of rRABVs to assess the impact of rLBNSE-GitrL on the pathogenicity in mice with an incompletely developed immune system, with survival rates monitored over 20 dpi. The data indicated no significant difference in survival between mice injected with rLBNSE-GitrL and those injected with rLBNSE (Figure 2B). Brain tissues from infected BALB/c mice were collected, and the levels of viral N mRNA (Figure 2C), vRNA (Figure 2D), and GitrL mRNA (Figure 2E) were measured using RT-qPCR at 6 dpi. No significant differences in N mRNA and vRNA levels were observed between rLBNSE-GitrL-infected and rLBNSE-infected mice at 6 dpi. However, GitrL mRNA expression was significantly higher in mice immunized with rLBNSE-GitrL compared to those immunized with rLBNSE. These findings suggest that rRABV-overexpressing GitrL in vivo does not impact rRABV transcription and replication in the central nervous system, nor does it compromise immune safety.

### 3.3. rLBNSE-GitrL Promotes Activation of DCs and Expression of IFN-α and IL-12p40 In Vitro

**Figure 3:**
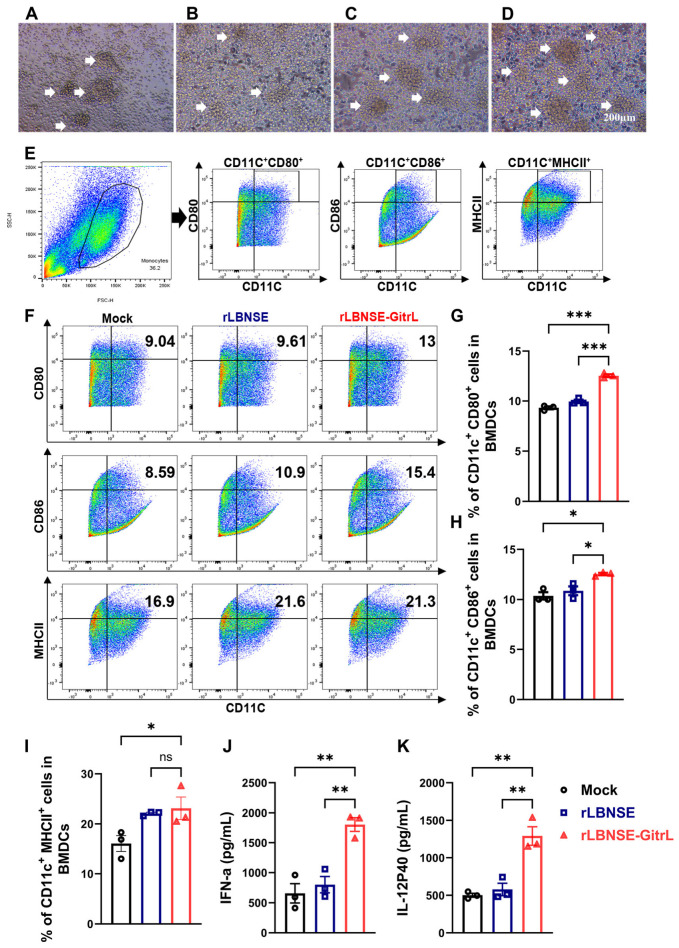
rLBNSE-GitrL promotes activation of DCs in vitro. (**A**) Isolation and cultivation of BMDCs. (**B**) DCs were cultured and stimulated with DMEM as the control group. (**C**) DCs were cultured and stimulated with rLBNSE. (**D**) DCs were cultured and stimulated with rLBNSE-GitrL. The white arrow indicated clustered DCs. (**E**) Gating strategies for the detection of the mature CD11C^+^CD80^+^BMDCs, CD11C^+^CD86^+^BMDCs, and CD11C^+^MHCII^+^BMDCs. (**F**) Representative flow cytometry plots of activated DCs. (**G**) Percentage of activated DCs (CD11C^+^CD80^+^) per 10^5^ BMDCs after 24 h of incubation. (**H**) Percentage of activated DCs (CD11C^+^CD86^+^) per 10^5^ bone BMDCs after 24 h of incubation. (**I**) Percentage of activated DCs (CD11C^+^MHCII^+^) per 10^5^ BMDCs after 24 h of incubation. (**J**) The expression level of IFN-α in the supernatant of activated DCs was detected by ELISA. (**K**) The expression level of IL-12p40 in the supernatant of activated DCs was detected by ELISA. The error bar represents the mean ± SD (n = 3). The following symbols are used to indicate significant differences between groups: *, *p* < 0.05; **, *p* < 0.01; ***, *p* < 0.001; ns, not significant.

To assess whether rLBNSE-GitrL facilitates the maturation of BMDCs in vitro, DCs were isolated from BALB/c mice and cultured with DMEM, rLBNSE, and rLBNSE-GitrL at a MOI of 1. Following a 24 h incubation, rLBNSE-GitrL prompted the aggregation of DCs into more cell clusters (Figure 3A–D). Flow cytometry was conducted to evaluate DC activation, as indicated by markers CD11C^+^CD80^+^, CD11C^+^CD86^+^, and CD11C^+^MHCII^+^ (Figure 3E,F). The results demonstrated that rLBNSE-GitrL significantly enhanced the activation of DCs characterized by CD11C^+^CD80^+^, CD11C^+^CD86^+^, and CD11C^+^MHCII^+^ markers in vitro (Figure 3G–I). Prior research indicates that activated DCs can secrete IFN-α [16] and IL-12p40 [17], thereby augmenting innate immune responses. The expression levels of IFN-α and IL-12p40 in the DCs culture supernatant were measured using ELISA after a 24 h incubation period. Results demonstrated that rLBNSE-GitrL induced activated DCs to produce higher levels of IFN-α and IL-12p40 compared to the parental rLBNSE (Figure 3J,K). This indicates that rLBNSE-GitrL enhances DCs activation and the subsequent secretion of IFN-α and IL-12p40 following rRABVs immunization.

### 3.4. rLBNSE-GitrL Increases Number of Activated DCs In Vivo

**Figure 4:**
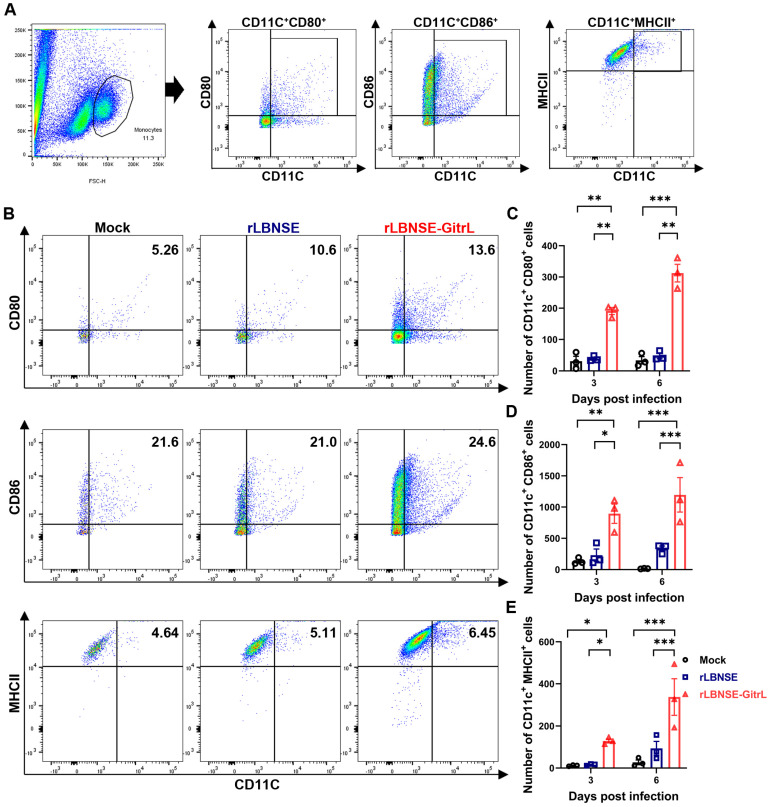
rLBNSE-GitrL increases the number of activated DCs in vivo. (**A**) Cell populations and gating strategies for DCs by flow cytometry within LNs. (**B**) Representative flow cytometry plots of activated DCs. (**C**) Total number of activated DCs (CD11C^+^CD80^+^) per 10^5^ draining LNs cells at 3 and 6 dpi. (**D**) Total number of activated DCs (CD11C^+^CD86^+^) per 10^5^ draining LNs cells at 3 and 6 dpi. (**E**) Total number of activated DCs (CD11C^+^MHCII^+^) per 10^5^ draining LNs cells at 3 and 6 dpi. The error bar indicates the mean ± SEM (n = 3). The following symbols are used to indicate significant differences between groups: *, *p* < 0.05; **, *p* < 0.01; ***, *p* < 0.001.

Several lines of evidence have shown that DCs are critical for the establishment of optimal innate immune responses after RABV immunization [18]. To assess whether the expression of GitrL by rRABV facilitates DC activation in vivo, we administered an intramuscular injection of 100 µL (10^6^ FFU) of rLBNSE-GitrL, the parent virus rLBNSE, or DMEM as a mock control, into 6-week-old mice. Lymph nodes were harvested and processed into single-cell suspensions (10^5^ cells) at 3 and 6 dpi. The activation of DCs (CD11C^+^CD80^+^, CD11C^+^CD86^+^, and CD11C^+^MHCII^+^) in the LNs was assessed by flow cytometry (Figure 4A). Representative flow cytometry data for DCs are shown (Figure 4B). Mice immunized with rLBNSE-GitrL exhibited significantly higher frequencies of activated DCs compared to those immunized with rLBNSE at 3 and 6 dpi (Figure 4C–E). These findings suggest that the overexpression of GitrL promotes the activation of DCs following rRABV immunization.

### 3.5. Transcriptome Sequencing and Differentially Expressed Gene Analysis

**Figure 5:**
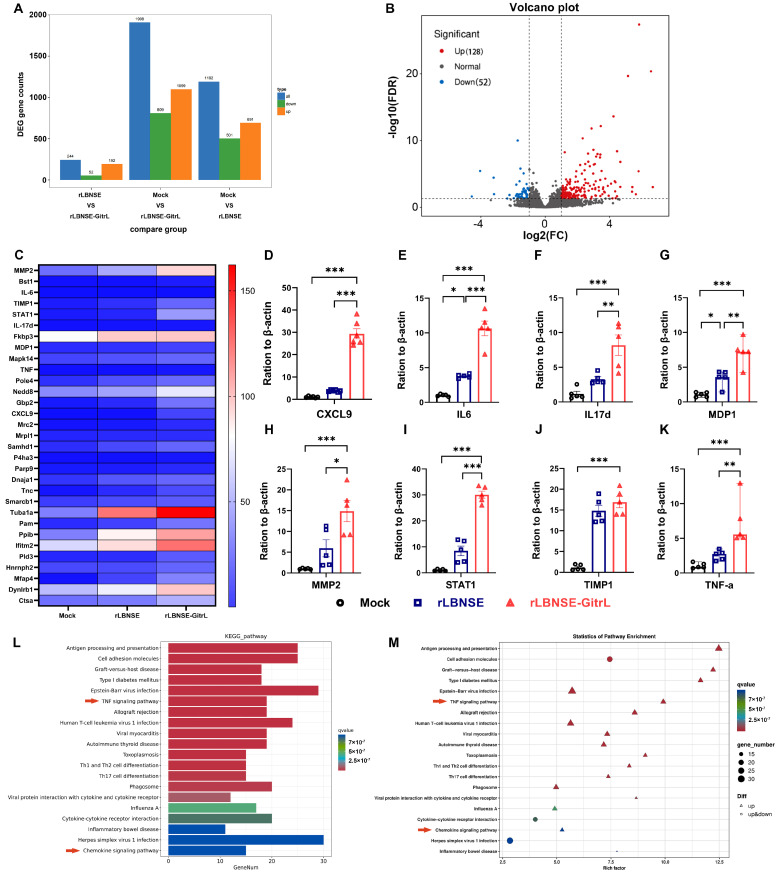
Transcriptome sequencing and differentially expressed gene analysis. (**A**) Comparative bar chart of differential genes. (**B**) Volcano plot of the differentially expressed genes between immunized mice of rLBNSE and rLBNSE-GitrL. (**C**) Gene expression heatmap. (**D**) Validation of the CXCL9. (**E**) Validation of the IL-6. (**F**) Validation of the IL-17d. (**G**) Validation of the MDP1. (**H**) Validation of the MMP2. (**I**) Validation of the STAT1. (**J**) Validation of the TIMP1. (**K**) Validation of the TNF-α. (**L**) Bar chart of KEGG enrichment pathway of the differentially expressed genes between rLBNSE and rLBNSE-GitrL. (**M**) KEGG enrichment pathway scatter plot of the difference expression genes between rLBNSE and rLBNSE-GitrL. The error bar indicates the mean ± SEM (n = 3). The following symbols are used to indicate significant differences between groups: *, *p* < 0.05; **, *p* < 0.01; ***, *p* < 0.001.

Transcriptomic sequencing analysis revealed 1908 differentially expressed genes in mice immunized with rLBNSE-GitrL compared to DMEM controls, comprising 1099 upregulated and 809 downregulated genes. Furthermore, mice immunized with rLBNSE-GitrL exhibited 244 differentially expressed genes, including 192 upregulated and 52 downregulated, relative to those immunized with rLBNSE (Figure 5A,B). This suggests that the rRABV expressing GitrL enhances differential gene expression.

The expression of significantly upregulated genes was analyzed using heatmap visualization. This analysis was conducted on gene expression data obtained from comparison of libraries derived from mice immunized with rLBNSE-GitrL, rLBNSE, and DMEM. The highly expressed genes identified include CXCL9, IL-6, IL-17d, MDP1, MMP2, STAT1, TIMP1, and TNF-α, among other cytokines (Figure 5C). These cytokines are associated with the innate immune response. To validate the RNA-sequencing results, the expression levels of selected upregulated cytokines were further quantified using RT-qPCR (Figure 5D–K).

The differentially expressed genes identified through comparative analysis of gene expression libraries from mice immunized with rLBNSE-GitrL, rLBNSE, and DMEM were subjected to KEGG pathway enrichment analysis. The results revealed that, in comparison to the rLBNSE group, the rLBNSE-GitrL group exhibited upregulation of the TNF, and chemokine signaling pathway (Figure 5L,M).

### 3.6. Overexpression of GitrL Improves Antibody Response and Protection Against Pathogenic RABV Challenge

**Figure 6:**
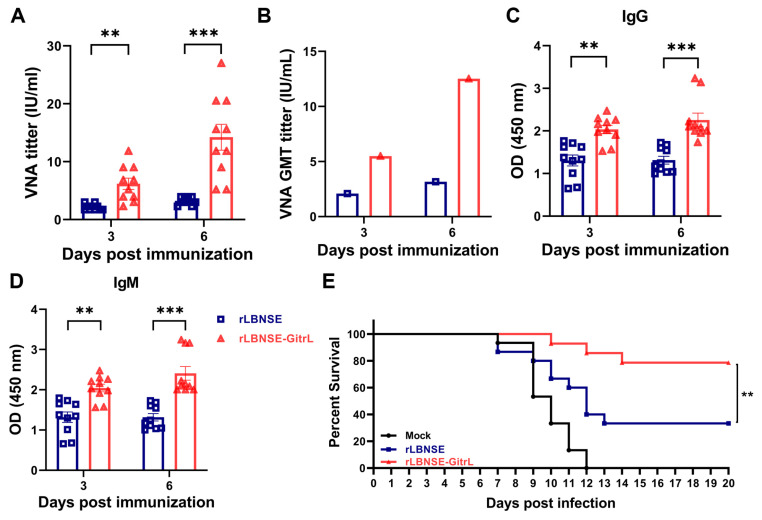
Overexpression of GitrL improves antibody response and protection against pathogenic RABV challenge. ICR mice (n = 10) were i.m. vaccinated with 100 µL volume containing 10^6^ FFU rLBNSE or rLBNSE-GitrL, or an equivalent amount of DMEM (as mock). Serum samples were collected at 3 and 6 dpi, and VNA titers (**A**) and GMT (**B**) were measured by FAVN assay. (**C**,**D**) The OD values of RABV G-specific IgG (**C**) and IgM (**D**) in the serum of immunized mice were determined by ELISA. (**E**) At 3 dpi, groups of immunized ICR mice (n = 15) were challenged with 50 LD50 of CVS-24 via i.c. route, and then monitored for another 20 days, and survivorship was recorded. The error bar indicates the mean ± SEM. The following symbols were used to indicate significant differences between groups: **, *p* < 0.01; ***, *p* < 0.001.

To evaluate whether the activation of DCs and modulation of innate immune signaling pathways rapidly enhance the immunogenicity of rLBNSE-GitrL, we intramuscular injection immunized 6-week-old female ICR mice (n = 10) with either rLBNSE (10^6^ FFU), rLBNSE-GitrL (10^6^ FFU), or DMEM (as mock). Serum samples were collected from the mice at a specific point in time, and VNA levels were assessed using the FAVN test. The data revealed that mice vaccinated with rLBNSE-GitrL exhibited significant VNA levels in their serum at 3 and 6 dpi, suggesting that the rRABV rapidly induces VNA production, due to GitrL expression (Figure 6A). The rLBNSE-GitrL-immunized mice exhibited a rapid and substantial increase in GMT, reaching 5.49 IU/mL at 3 dpi and 12.51 IU/mL at 6 dpi. In contrast, the rLBNSE-immunized mice displayed GMT values of 2.10 IU/mL at 3 dpi and 3.17 IU/mL at 6 dpi (Figure 6B). ELISA analysis revealed that the rLBNSE-GitrL immunization elicited significantly elevated levels of both IgG (Figure 6C) and IgM (Figure 6D) against the rabies virus glycoprotein (RABV G) at 3 and 6 dpi, as expected.

To further investigate the relationship between early antibody production and protection, we evaluated the efficacy of rLBNSE-GitrL against lethal rabies virus (RABV) challenge. At 3 dpi, mice were intracranially challenged with 50 times the lethal dose (50 × LD50) of the CVS-24 RABV strain and monitored daily for 20 additional days. All mice in the DMEM (as mock) group succumbed to the infection within 14 days post-challenge. The data demonstrate a significantly higher percentage of survivor ratios in mice vaccinated with rLBNSE-GitrL (86.67%) compared to those vaccinated with rLBNSE (33.33%) (Figure 6E). These findings suggest that the overexpression of GitrL in rRABV can substantially enhance the early antibody response and provide effective protection.

## 4. Discussion

Our research demonstrates that rLBNSE-GitrL activates DCs and modulates innate immune signaling pathways, thereby enhancing innate immunity following RABV immunization. Compared to rLBNSE, the rRABV expressing GitrL significantly increased VNA production as early as 3 dpi, leading to earlier and more effective protection against pathogenic RABV challenge. Previous studies have demonstrated that recombinant RABV expressing cytokines like IL-7 [19], IL-21 [20], and CXCL13 [21,22] enhances immunogenicity through primary germinal center responses. However, these studies report relatively slower antibody responses compared to our findings. Our data indicate that the activation of dendritic cells and modulation of innate immune-related signaling pathways are crucial for augmenting early RABV immunogenicity and protection.

The foremost criterion in vaccine development is safety. In this study, the rLBNSE-GitrL titer and the sequence of the inserted GitrL were stably subcultured for at least ten consecutive passages. Based on growth curves, average fluorescence spot density, cell viability in vitro, and body weight changes, survival rates, and virulence in vivo, concerns regarding the safety of using rLBNSE-GitrL as a live-attenuated rabies vaccine can be alleviated. The pathogenicity experiment revealed that mice infected with rLBNSE-GitrL exhibited a slight, statistically insignificant weight loss at 2 days post-infection, potentially attributable to an inflammatory response [23]. Notably, there were no significant differences in the survivor ratios between the rLBNSE-GitrL-infected and rLBNSE-infected mice. In addition, the parental strain rLBNSE was derived from mutations at amino acid positions 194 and 333 of the G proteins in the SAD-B19 strain. This rLBNSE strain is non-pathogenic when administered intramuscularly or orally as an animal vaccine [24,25]. These results suggest that rLBNSE-GitrL holds potential as a live-attenuated and uninjurious rabies vaccine for animals.

Unlike other TNF superfamily members, GitrL is expressed on resting APCs [26], its increased expression on these resting APCs suggests a role in linking innate immune recognition with the adaptive immune response to viruses, specifically through a CD8^+^ T cell-dependent pathway [27], and facilitating early T cell activation [28]. The bidirectional signaling of GitrL can stimulate mouse plasma cells like DCs to produce substantial amounts of type I IFNs, such as IFN-α, thereby inducing an early protective response against viruses [29]. The GitrL pathway is known to facilitate the migration of activated DCs from the local site to the LNs [30]. This process may promote the proliferation of activated DCs within the LNs. Following DC activation, GitrL expression is transiently upregulated [31]. Enhanced GitrL interaction with its receptor has been shown to improve resistance to viral infection in animal studies, analogous to the effects of OX40, another member of the tumor necrosis factor receptor superfamily [32]. This suggests that the GitrL signaling pathway may play a crucial role in the protective immune response against rabies. Correspondingly, the upregulation of IFN-α [16] and IL-12p40 [17] may enhance innate immune responses. Consequently, costimulation through GitrL expressed by rLBNSE-GitrL effectively increased the proliferation of activated and mature DCs both in vitro and in vivo. This process also elevated the secretion of IFN-α and IL-12p40 by activated DCs, leading to higher levels of VNA and superior protection compared to the parent virus as early as 3 dpi.

Our results demonstrate that GitrL overexpression activates the innate immune response to RABV and enhances antibody production following rRABV vaccination. We hypothesized that GitrL would confer increased protection during RABV immunization. Indeed, mice immunized with rLBNSE-GitrL exhibited greater resistance to RABV challenge. In particular, we observed that mice immunized with rLBNSE-GitrL exhibited enhanced activation of innate immune signaling pathways, antiviral genes, and inflammatory mediators compared to those immunized with the parent virus. Prior research indicates that local inflammation promotes interactions between infected and immune cells, boosts antibody production [33], and aids in virus clearance [34]. The present investigation demonstrated that GitrL overexpression amplifies the previous activation of TNF and chemokine signaling pathways and moderately upregulates the expression of innate immune-related genes, such as MMP2, IL-6, CXCL9, STAT1, IL-17d, TNF-a, and MDP1, following rRABV vaccination compared to the parent virus. PAMPs of invading microorganisms are detected and bound by various PRRs in the host, including TLRs, NLRs, and RLRs [35]. The interaction between TLRs and TIR domain-containing receptors initiates a downstream signaling cascade that reactivates the TNF [36], and chemokine [37], signaling pathways and upregulates co-stimulatory molecules on DCs [38]. These findings indicate that rLBNSE-GitrL vaccination effectively activates several innate immune signaling pathways, enhances the production of innate immune-related inflammatory factors, and facilitates the early induction of protective antibody responses.

## 5. Conclusions

In conclusion, our study shows that the rRABV expressing GitrL effectively enhances innate immunity by activating DCs and innate immune signal pathways and genes, leading to a swift and strong protective antibody response. This rLBNSE-GitrL could be developed into an effective and nonpathogenic animal vaccine.

## Figures and Tables

**Table 1:** Primers for RT-qPCR.

Primers	Sequence (5′–3′)
β-actin-F	CACTGCCGCATCCTCTTCCTCCC
β-actin-R	CAATAGTGATGACCTGGCCGT
N mRNA-F	GATCGTGGAACACCATACCC
N mRNA-R	TTCATAAGCGGTGACGACTG
vRNA-F	CTCCACAACGAGATGCTCAA
vRNA-R	CATCCAACGGGAACAAGACT
GitrL-F	GGGCAGAGAGGTGCAAGAAG
GitrL-R	CTTCAGCTTCCCATCAGATG
STAT1-F	GGCCTCTCATTGTCACCGAA
STAT1-R	TGAATGTGATGGCCCCTTCC
CXCL9-F	GGAGTTCGAGGAACCCTAGTG
CXCL9-R	GGGATTTGTAGTGGATCGTGC
TIMP1-F	AGAGACACACCAGAGCAGATACC
TIMP1-R	AGCCCTTATGACCAGGTCCG
MMP2-F	TTCCCTAAGCTCATCGCAGACT
MMP2--R	CACGCTCTTGAGACTTTGGTTCT
IL-6-F	ACAGAAGGAGTGGCTAAGGA
IL-6-R	CGCACTAGGTTTGCCGAGTA
IL-17d-F	GGATTTCCTACGACCCTGCTC
IL-17d-R	CCGGGATGGTGATGTAGTGTTC
MDP1-F	TGCCAAGTAGCCACATCGAG
MDP1-R	GAGATGGGGGTTGAGGAACG

## Data Availability

The original data presented in the study are openly available in Science Data Bank at https://doi.org/10.57760/sciencedb.29425.
